# Application of an innovative biventricular assist device for bridge-to-transplant in a low-weight child with end-stage restrictive cardiomyopathy

**DOI:** 10.1016/j.xjtc.2026.102419

**Published:** 2026-05-14

**Authors:** Xiaocheng Liu, Xuming Mo, Zhengqing Wang, Wei Peng, Shutang Ren, Shengfu Su, Shifu Wang, Liang Hu, Xiujuan Zhang, Jirong Qi, Wei Wang, Shan Zhong, Kegang Jia, Yahong Yu, Feng Chen, Rui Jing, Hongwu Wang, Lijuan Fan, Jianming Li

**Affiliations:** a Department of Cardiac Surgery, Nanjing Children's Hospital, Nanjing Medical University, Nanjing, China; b Department of Cardiovascular Surgery, TEDA International Cardiovascular Hospital, Tianjin University, Tianjin, China

**Figure:**
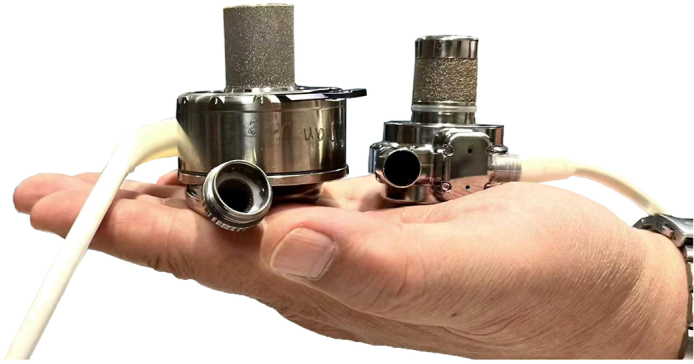
The novel pediatric ventricular assist device-HeartCon II.

**Central Message**

We discuss the successful implantation of the world's lowest body-weight pediatric biventricular intracorporeal assist devices (HeartCon II) for end-stage restrictive cardiomyopathy.

Implantation of biventricular assist devices (BiVADs) for a bridge-to-transplantation in a young girl weighing 12.2 kg with end-stage restrictive cardiomyopathy (RCM) was performed at Nanjing Children's Hospital in August 2025. The patient subsequently underwent successful allogeneic heart transplantation at TEDA International Cardiovascular Hospital on postoperative day 40. To our knowledge, this is the world's lowest body-weight case of pediatric BiVAD implantation with the use of magnetic levitated ventricular assist devices for end-stage heart failure (ESHF) caused by RCM. The patient was in excellent condition upon postoperative 6-month follow-up at our outpatient clinic. Our clinical experience, with the technological breakthroughs, is summarized herein.

## Case Report

A 5-year, 3-month-old girl presented with severe growth retardation, decreased activity tolerance, and progressively worsening ascites over the preceding 3 years. The volume of ascites increased rapidly within 3 months before her admission. Findings of the physical examination revealed her abdominal circumference was 74 cm, with abdominal wall venous engorgement and a protruding umbilicus. Her arterial oxygen saturation was 90%, due to right to left shunt through a patent foramen ovale. She had severe shortness of breath even after minimal activity, with a respiratory rate persistently greater than 28 breaths per minute. The child preferred squatting and experienced dyspnea whenever lying flat. She was born at full term with a birth weight of 3 kg, had no family history of RCM, and genetic testing revealed no abnormalities.

This study was approved by the TEDA International Cardiovascular Hospital Ethics Committee (no. 202508018-2) on August 20, 2025, and written informed consent was obtained from the patient's legal guardian for publication of this case report and accompanying images.

## Auxiliary Examinations

**Figure E1:**
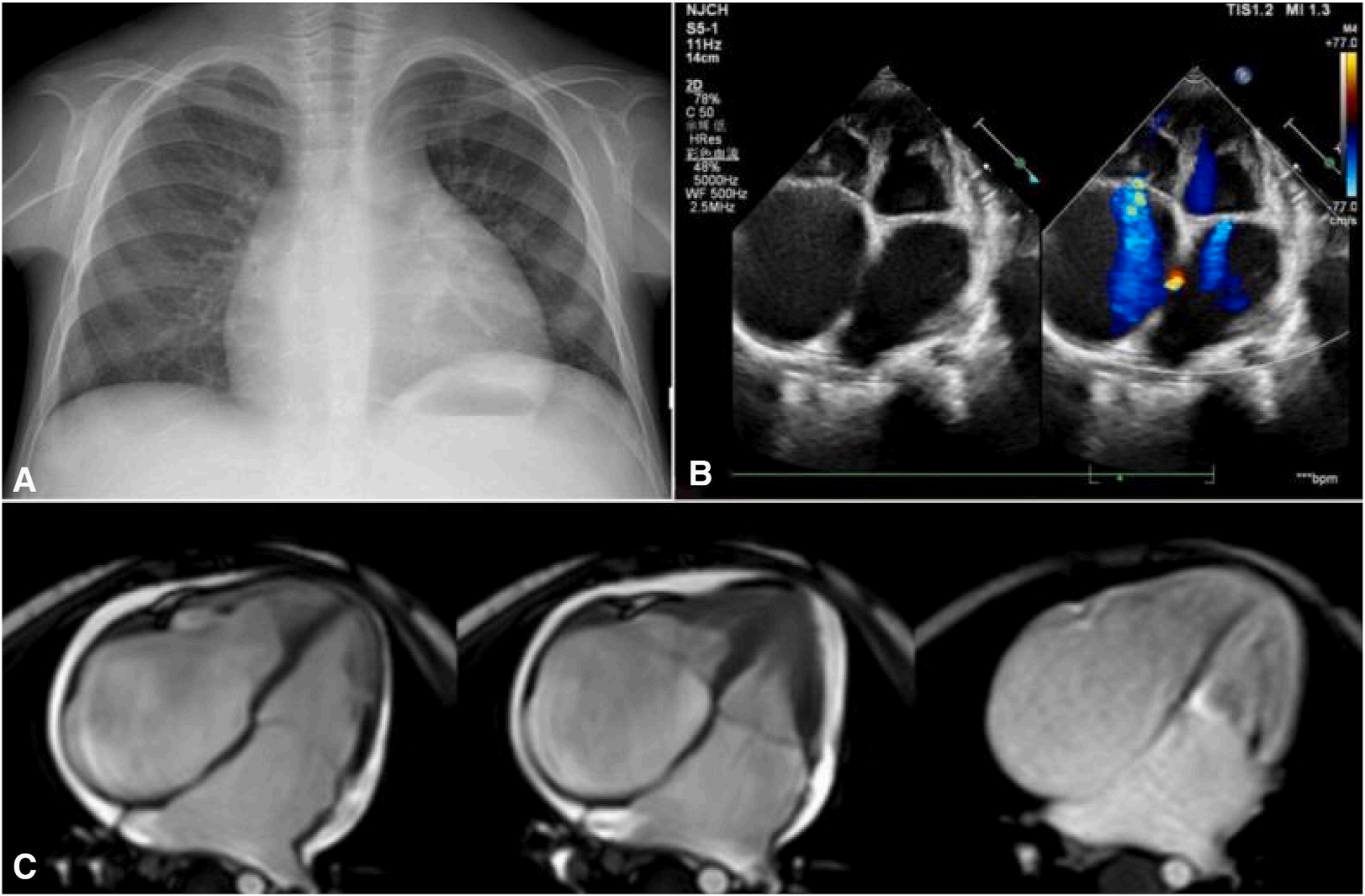
Preoperative imaging examinations. A, Radiograph of the chest. B, Echocardiography. C, Cardiac magnetic resonance.

Radiograph of the chest showed pulmonary congestion and cardiomegaly (Figure E1, *A*). Echocardiography revealed significant bilateral atrial enlargement (predominantly in the right atrium) with bilateral small ventricles (left ventricle: 44 mm in length, 27 mm in transverse diameter; right ventricle: 33 mm in length, 26 mm in transverse diameter), which led to biventricular diastolic dysfunction. There was moderate mitral regurgitation and severe tricuspid regurgitation (Figure E1, *B*). An ultrasound scan of the abdomen confirmed congestive hepatomegaly with massive ascites. The maximal anteroposterior diameter of peritoneal effusion was 109 mm. Cardiac magnetic resonance showed extensive subendocardial delayed enhancement in the interventricular septum and left ventricular free wall, consistent with RCM (Figure E1, *C*). The left ventricular ejection fraction was 80%, left ventricular end-diastolic volume index was 37.6 mL/m^2^, and left ventricular end-systolic volume index was 7.4 mL/m^2^; The right ventricular ejection fraction was 64%, right ventricular end-diastolic volume index was 45.2 mL/m^2^, and right ventricular end-systolic volume index was 16.4 mL/m^2^.

## Multidisciplinary Therapeutic Strategies

**Figure 1:**
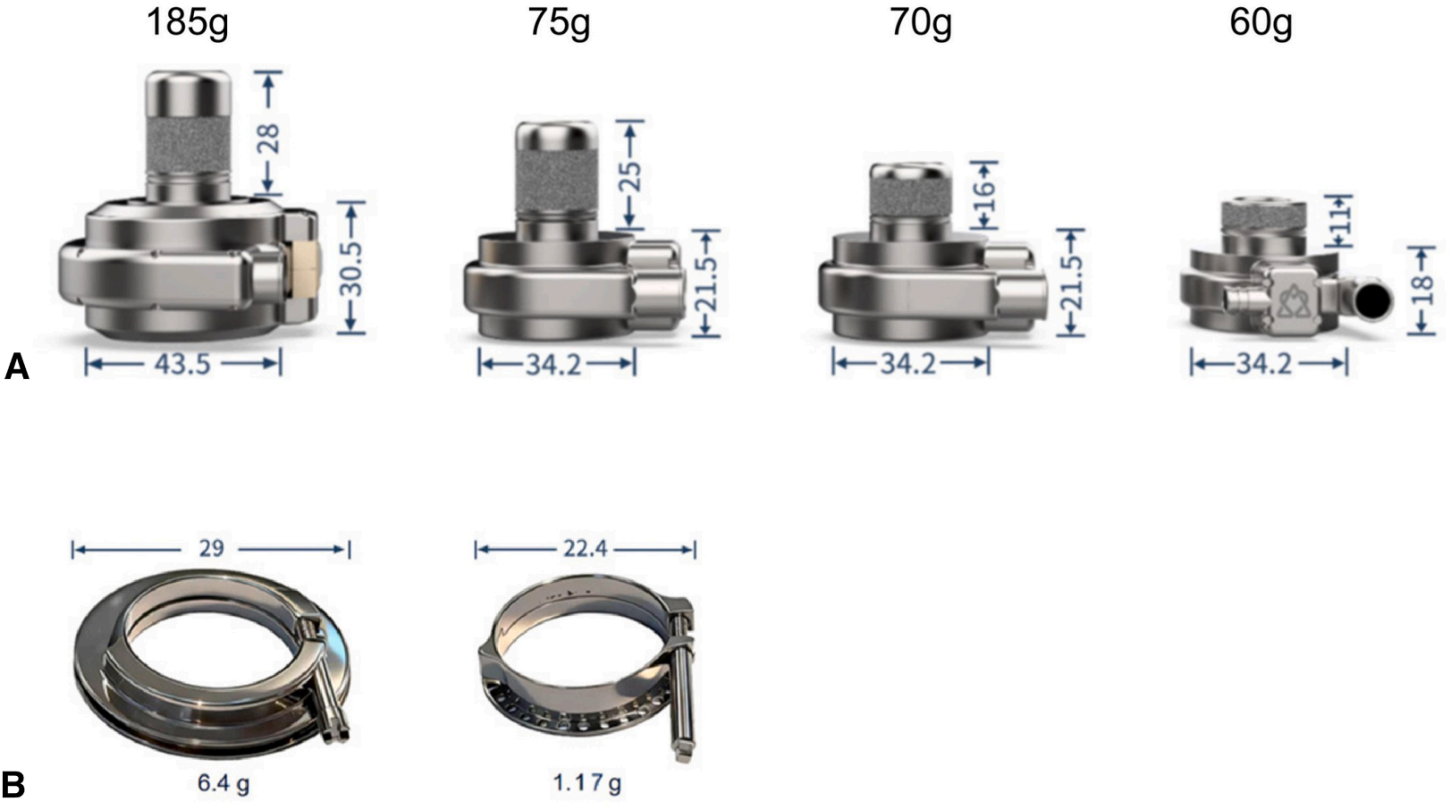
Series products of the “Rocket HC VAD Family.” A, HC I, HC Ⅱ, HC Ⅱ-1, and HC Ⅱ-2 (from *left* to *right*): HC I is a hydromagnetic levitation VAD; HC Ⅱ series are fully magnetic-levitation VADs. B, Comparison of the standard HC Ⅱ-1 sewing ring (*left*) and custom-made sewing ring (*right*). *VAD*, Ventricular assist device.

The child was diagnosed with ESHF attributable to RCM, and heart transplantation (HTx) was indicated. In view of the donor organ shortage and the patient's life-threatening condition, BiVAD implantation was chosen as a bridge-to-transplantation. Because no commercially available ventricular assist devices (VADs) are specifically designed for biventricular mechanical support in children, the engineering team reduced the size and weight of the existing HeartCon II device, a fully magnetically-levitated VAD (HC II; ROCOR Medical Technology Co, Ltd) without active magnetic bearings or position sensors in the pump. To adapt to the child's small thoracic and cardiac cavities, the length of the HC II inflow cannula was reduced from 25 mm to 16 mm and 11 mm for left ventricular and right atrial implantation, respectively. The total pump weight was reduced from 75 g (HC II) to 70 g (HC II-1), and further to 60 g (HC II-2; Figure 1, *A*), respectively. The outer diameter of the sewing ring was reduced from 29 mm to 22.4 mm, and its weight was reduced from 6.4 g to 1.17 g, to fit the extremely small surface area of the right atrium and the left ventricular apex (Figure 1, *B*).

## Surgical Technique and Intraoperative Management

The child was 5 years, 3 months old at surgery. Her body weight was 18 kg (with massive ascites), height was 100.5 cm, with a body surface area of 0.60 m^2^ and body mass index of 12.9 kg/m^2^. At the initiation of anesthesia, central venous pressure (CVP) was 29 mm Hg. Ascites was drained in 100-mL increments, guided by transesophageal echocardiography and circulatory parameter monitoring to ensure hemodynamic stability. A total of 5000 mL of ascites was slowly drained. Her body weight reduced to 12.2 kg postdrainage (ie, reflecting severe growth retardation equivalent to the level of a normal 2-year-old child). Her CVP was normalized after ascites drainage.

**Figure 2:**
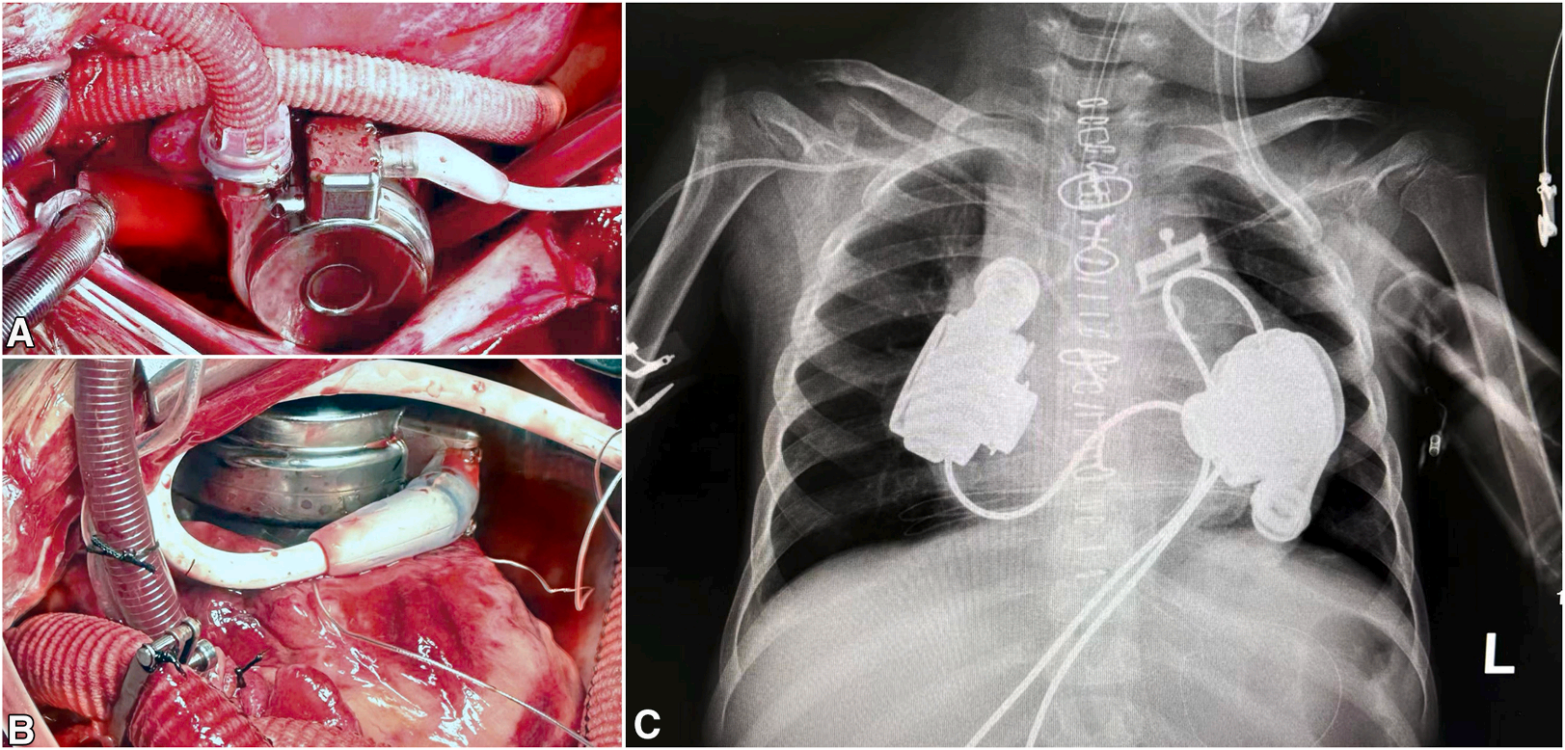
Implantation of LVAD and RVAD. A, An LVAD was implanted into the left ventricle and a custom-made flow restrictor was placed on the outflow graft of the RVAD. B, An RVAD was implanted in the right atrium with the LVAD outflow conduit being routed under the RVAD outflow conduit. C, Postoperative radiograph of the chest. *LVAD*, Left ventricular assist device; *RVAD*, right ventricular assist device.

After standard median sternotomy, cardiopulmonary bypass was established. Ventricular fibrillation was induced. The right atrium (RA) was opened, and the patent foramen ovale was closed. The mini sewing ring was sutured at the level of the crista terminalis in the midportion of the dilated RA. The RA wall within the ring was excised, and suturing was completed to fix the RA cut edge to the sewing ring from inside of the atrium to prevent possible floating tissue obstructing the ultra-short HC II-2 inflow cannula. The pump inflow cannula was inserted and the right VAD was implanted into the RA (Figure 2 and Video 1). A continuous mattress suture was completed to longitudinally reduce the diameter of the 10-mm artificial vessel graft to 7 mm (funnel-shaped), matching the small caliber of the pulmonary artery. The distal end of the artificial conduit was anastomosed to the pulmonary artery (PA) with a reverse “S” shape (Figure 2 and Video 1). Two to three stitches at the distal anastomosis were left untightened for rapid deairing after pump activation.

**Figure E2:**
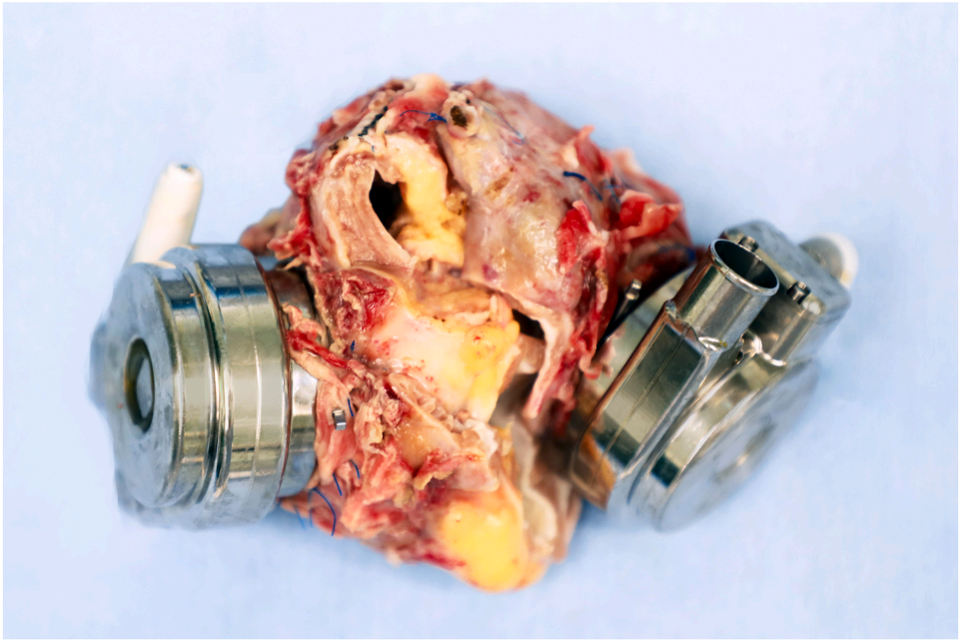
Gross specimens of the diseased heart (excised after transplantation) and the biventricular assist device.

**Figure E3:**
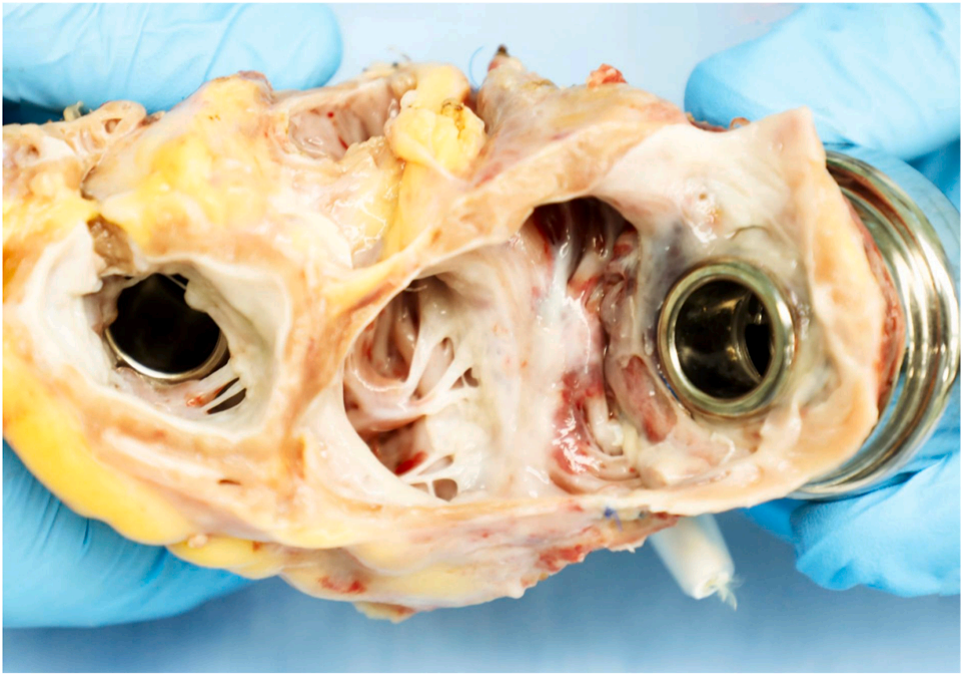
Gross specimen viewed from inside of the atria shows that the end of the LVAD inflow cannula reaches the edge of the MV (*left*); the RVAD inflow cannula protrudes only 1 mm into the RA (*right*), with smooth surrounding atrial endocardium; the tricuspid valve is in the middle. *LVAD*, Left ventricular assist device; *MV*, mitral valve; *RVAD*, right ventricular assist device; *RA*, right atrium.

After heart transplantation, the excised heart specimen revealed that, although the 2 VADs are small, the RCM heart remained disproportionately small (Figure E2). Examination of the RA specimen showed that the RVAD's inflow cannula extended only 1 mm into the RA cavity, with a smooth surrounding surface (Figure E3).

The LV transverse diameter was only 27 mm, and the axial dimension was only 44 mm. To prevent the pump inflow cannula being sucked into the small ventricle, the myocardial tissue in the distal LV (ie, from the papillary muscle root to the apex, including the interventricular septum) was uniformly thinned to 4 mm in thickness, significantly enlarging the distal ventricular cavity. The mini-sewing ring was fixed to the apical drilling site (Figure 2 and Video 1).

**Figure E4:**
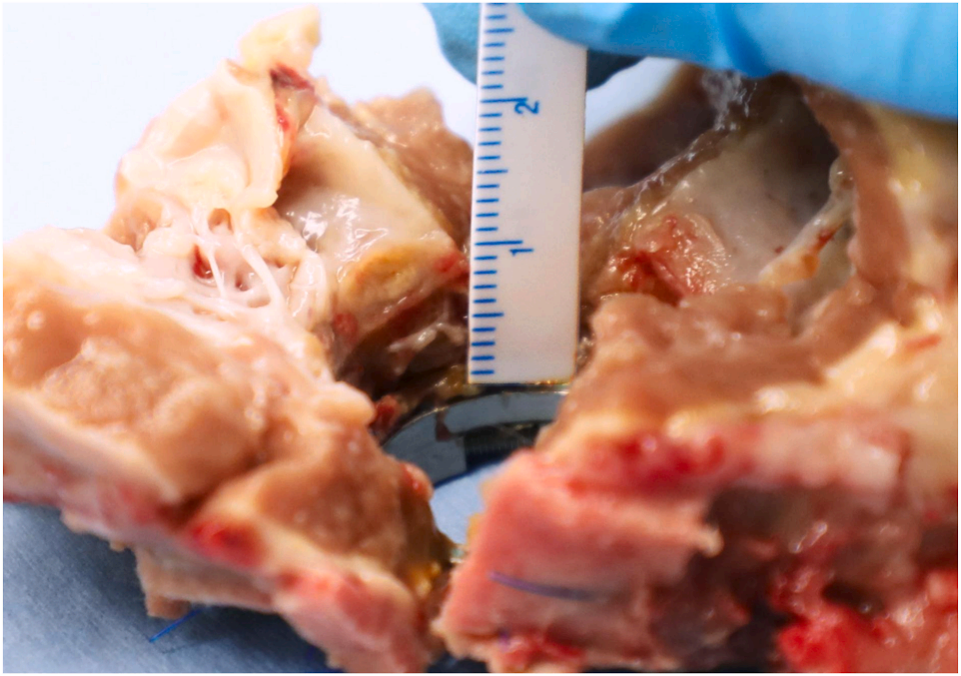
The inflow cannula protrudes only 6 mm into the left ventricular cavity, with smooth surrounding endocardium.

**Figure E5:**
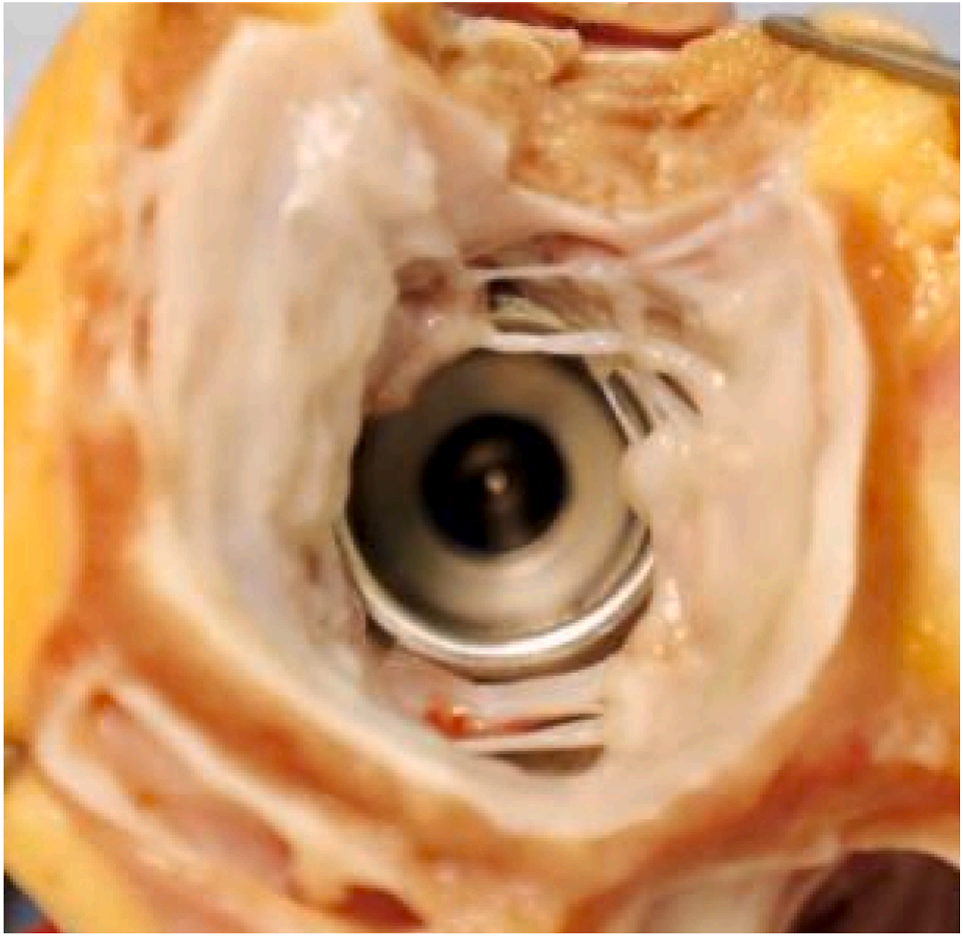
Left ventricular assist device inflow cannula reaches the edge of the mitral valve.

The left ventricular assist device (LVAD) inflow cannula was inserted into the left ventricle (LV) cavity. The artificial conduit was routed under the right pump graft, and the caliber-reduced 7-mm-diameter graft was anastomosed to the ascending aorta. The transplanted heart specimen showed that the HC II-1 inflow cannula protruded into the left ventricle by only 6 mm (Figure E4). When viewed from the LA, the inflow cannula has already reached the edge of the mitral valve (Figure E5).

The superior and inferior venae cavae clamps were released, and the heart resumed spontaneous beating. After we removed the CVP catheter, the right pump was activated at 1400 rpm. The left pump was activated at 2200 rpm. After deairing, the aortic and pulmonary side-biting clamps were released. Both pumps operated normally.

## Intraoperative Adjustment

Three catheters were placed through the chest wall: one via the interatrial groove to measure left atrial pressure (LAP), and 2 triple-lumen ones to monitor the PA pressure and for fluid infusion. Transesophageal echocardiography was used to assess cardiac volume and biventricular balance, targeting the mean arterial pressure (MAP) to the mean pulmonary artery pressure (MPAP) at a ratio of 4-5. Although the speed of HC Ⅱ-3 has been reduced to the minimum, its unloading was still excessive relative to the child's low body weight. A custom-made flow restricting clamp was placed on the outflow graft to precisely reduce the cross-sectional area of the graft until the target MAP:MPAP ratio was achieved.

**Figure 3:**
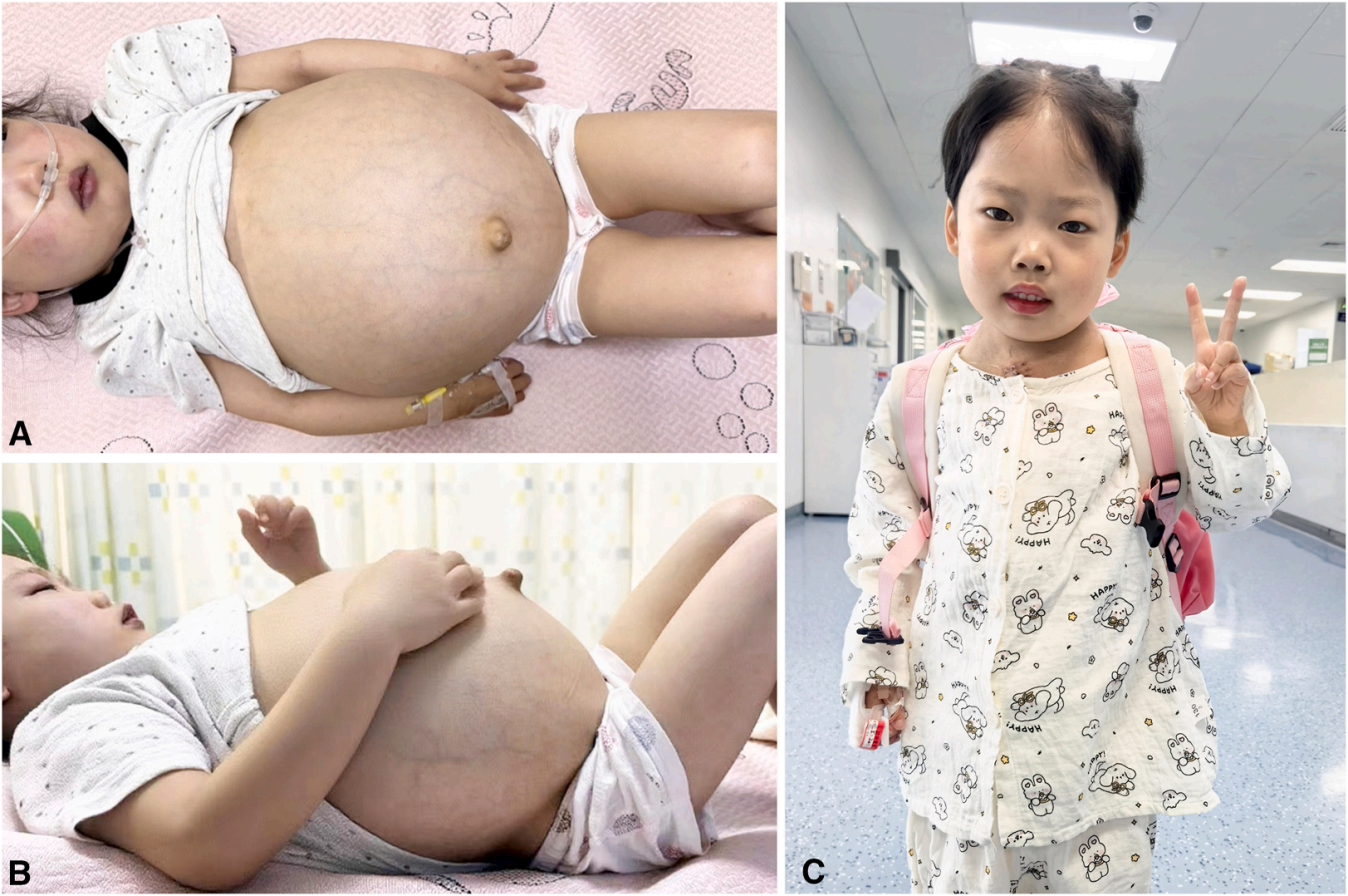
Pre- and postoperative comparison of the child (with permission). A and B, Preoperative. C, Postoperative day 10.

A radiograph of the chest taken postoperatively showed that the modified BiVAD did not compress the heart or lungs (Figure 2, *C*). The child was ambulatory on postoperative day 5 (Figure 3, *A* and *B*, shows her preoperative condition and Figure 3, C, shows the child on postoperative day 10). Her physical strength improved significantly. The left atrial manometer tube was retained for 31 days, whereas the 2 pulmonary artery catheters were retained for 30 days and 33 days, respectively. All catheters were removed after thoracic closure, stabilized circulation, and removal of endotracheal intubation. We observed no difference in measurements between catheters inserted via these 2 approaches compared with those inserted via the jugular vein route, with consistent results observed after endotracheal intubation removal. Both approaches used an embedded tunnel design, and no bleeding occurred during removal. The child was transferred to TEDA International Cardiovascular Hospital on postoperative day 36 and underwent allogeneic HTx on postoperative day 40 successfully.

## Postoperative Challenges and Management

### Refractory Lymphatic Leakage and Nutritional Support

Complete resolution of severe right heart constraint led to refractory chyloperitoneum, with daily pleural effusion totaling up to 1395 mL. Cumulative ascites reached 13,274 mL by postoperative day 21, causing significant protein loss. Hence, albumin supplementation was administered to maintain colloid osmotic pressure at 25 mm Hg, with a total of 570 g albumin and 62.5 g immunoglobulin infused by postoperative day 25. Octreotide was given for 17.5 days to inhibit lymphatic leakage. Nutritional support was administered at >120 kcal/kg/d via oral and intravenous routes to achieve positive nitrogen and caloric balance in this severely cachectic child. One month after BiVAD implantation, her growth curve remained at the low limit of international standards, but she regained sufficient strength.

### Left and Right Pump Speed Regulation

In the context of hemodynamic fluctuations caused by massive serous fluid leakage, pump speeds were adjusted on the basis of ultrasound and hemodynamic parameters. We proposed a “left-before-right” strategy for pump speed adjustment:

“Two measures on the left”: Adjusting the left pump speed by closely monitoring the MAP and the LAP. While maintaining the MAP at 70 to 75 mm Hg and preventing LVAD suction, the pump speed was increased to minimize LAP, thereby reducing pulmonary vascular resistance and MPAP to achieve a MAP: MPAP at a ratio of 4-5.

“Six measures on the right”: Adjusting the right pump speed by monitoring 6 major hemodynamic parameters in the right heart system and ultrasound parameters: (1) Minimize MPAP to maintain the target MAP:MPAP ratio. (2) Minimize inferior vena cava diameter and optimize respiratory variability to reduce peritoneal leakage while controlling MPAP. (3) Ensure adequate RA filling, centralize the interatrial septum, and have RA:LA ratio >0.8. (4) Ensure adequate RV filling, have the interventricular septum to be centralized, straight or with concordant motion to the left ventricular posterior wall, and RV/LV ratio >0.6. (5) Ensure tricuspid valve opening with each heartbeat and absence of significant tricuspid insufficiency. Ensure pulmonary valve opening with each heartbeat and absence of significant pulmonary valve insufficiency, thereby minimizing the flow ratio of artificial conduit to PV (ie, maximizing RV blood flow).

### Adjustment of Heart Rate and Contractility to Prevent Pump Suction That May Be Caused by the Small Heart Chambers of RCM

**Figure E6:**
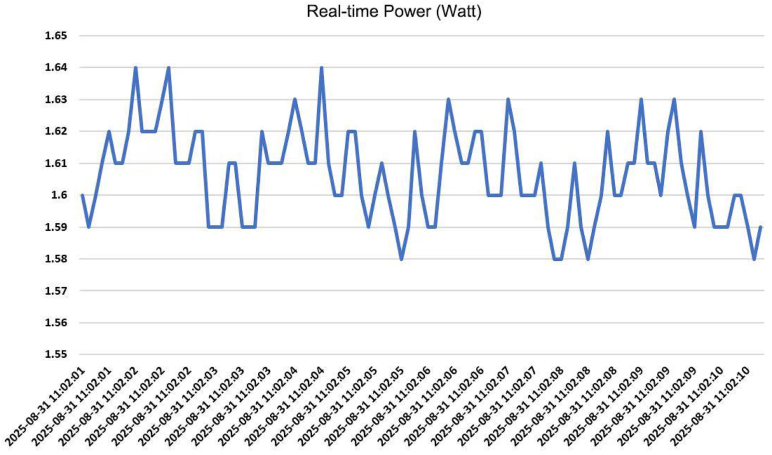
Real-time left ventricular assist device power waveform: regular suction waves appear in the power waveform during end-inspiration phase, periodical reduced left ventricular filling.

**Figure E7:**
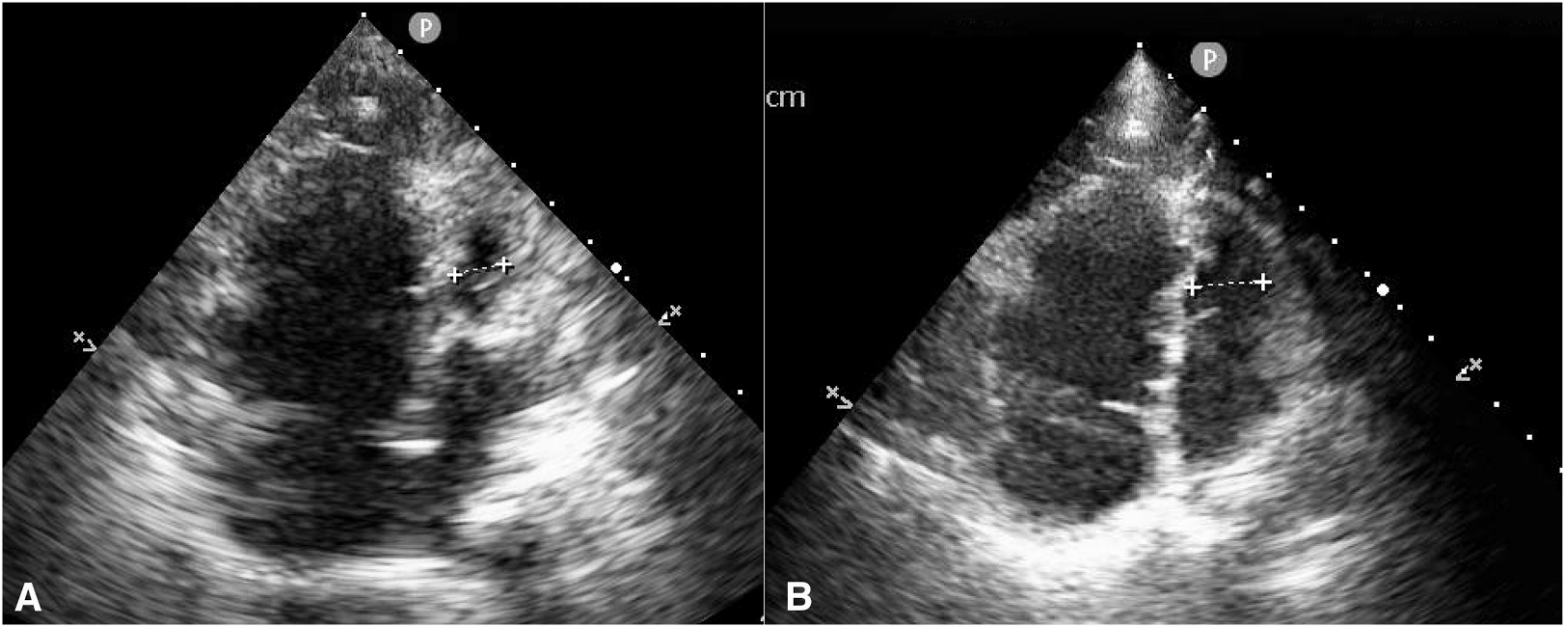
A, Triangular-shaped left ventricular cavity under ventricular assist device support. B, Tongue-shaped left ventricular cavity after administration of metoprolol and ivabradine.

Given the specific pathophysiological changes associated with RCM, managing interference with BiVAD assistance is of paramount importance. Increased LVAD unloading narrowed the LV into a triangular-shaped cavity, with the mitral valve as the base and the papillary muscle as the apex, and the distal LV chamber could no longer be clearly visualized. This resulted in a periodic suction wave in the power waveform of the LVAD in each respiratory cycle (Figure E6). It indicated that although the LVAD effectively emptied the small distal LV cavity (Figure E7, *A*), the 6-mm inflow cannula inserted into the LV did not cause ventricular collapse.

The combined use of metoprolol and ivabradine, administered to regulate contractility and heart rate (to <90 bpm), can restore the distal LV cavity from a triangular-shape to a tongue-shape (Figure E7, *B*), achieving proper biventricular balance under BiVAD support. In future practice, simply adjusting the blood pump inflow tube to protrude only 6 mm beneath the LV endocardium should prevent suction wall formation, as demonstrated by our multiple animal studies. On the basis of our experience, this approach is suitable for pediatric patients of low weight. Because shortening the blood pump inflow tube resolves suction issues, pediatric patients may avoid being switched to atrial cannulation or mitral valve removal. The successful implementation of inflow tube shortening in the second-generation EVAHEART further corroborates this conclusion.

## Discussion

For this patient with low weight and ESHF attributable to RCM and severe growth retardation, we successfully performed biventricular mechanical support using a specially modified implantable VADs. Under enhanced postoperative management, dealing with multiple complications, the child underwent HTx on postoperative day 40.

Global RVAD development has long been stagnant, resulting in very low implantation rates of BiVADs: 5.3% in adults (Intermacs 2024 Report^1^) and 2.8% in children (eighth Pedimacs Report^2^). From March 2012 to December 2024, the Advanced Cardiac Therapies Improving Outcomes Network (ACTION) registry recorded no cases of RCM supported by intracorporeal BiVAD.^3^ The unique anatomy of the right heart prevents existing LVADs from being used in children who are very small. Previous minimum age/weight records for implantable BiVADs were 4.0 years and 14 kg.^4^ Consequently, the paracorporeal Berlin Heart EXCOR was the only BiVAD used for bridge-to-transplantation in children with low weight. However, in China, the Berlin Heart EXCOR has not yet obtained clinical commercial approval, and it limits children's activity and has also been associated with a high risk of infection and bleeding.^5^^,^^E1^ For this purpose, we adopted this innovative approach to explore a fully implantable biventricular assist device for low-weight children, aiming to reduce the frequent complications of Berlin Heart EXCOR and improve their quality of life. This case is 1 of the 2 reported BiVAD implantations in ultra-small children with RCM,^4^ with a new record of the lowest body weight, to our knowledge. Customized modifications to the VADs and the accessories, and the engineering innovations in this case, are outlined to follow:

• The end of the 10-mm truncated HC Ⅱ-3 inflow cannula was nearly parallel to the RA endocardium; the 15-mm truncated HC Ⅱ-2 inflow cannula protruded only 6 mm into the LV, reaching the edge of the mitral valve without suctioning the severely narrowed LV cavity.
• Reducing the axial height of HC Ⅱ-1 to 37.5 mm for HC Ⅱ-2, and to 32.5 mm for HC Ⅱ-3 created the minimum axial heights of all the commercialized magnetically levitated centrifugal pumps, which minimizes wall suction risk and facilitates implantation into small thoracic cavities without cardiopulmonary compression.
• The reduced suture ring makes the weight and diameter of the pump and the ring more suitable for the anatomical characteristics of the chest and heart of young children. The BiVAD shares the same battery and controller, providing convenience for patients.

## Major Challenges in BiVAD Clinical Management

RCM causes small heart chambers in children, representing a significant challenge for VAD implantation. To create the necessary space, we uniformly thinned the hyperplastic LV free wall and septum into a cup-shape, significantly extending the volume from the papillary muscles to the apex. We also shortened the inflow cannulas of both VADs and downsized the sewing ring. Concurrently, combined medical therapies were used to reduce the heart rate and the contractility. These 3 modifications made it easier to fix the sewing ring on the extremely small heart and prevented cannular obstructions for successful ventricular unloading by the BiVAD.

LVAD unloading is antegrade because it is directly from the LV. The crescent-shaped anatomical cross-section of the RV, however, is not suitable for the placement of an RVAD. Therefore, blood pumps are often implanted into the right atrium. This type of unloading is a “short-circuit” that bypasses the right ventricle. Excessive unloading of the right heart not only induces iatrogenic pulmonary hypertension and biventricular imbalance, leading to pulmonic valve insufficiency and tricuspid insufficiency, but also results in right ventricular stasis, thrombus formation, and myocardial atrophy from disuse. For this reason, our approach is to minimize the amount of RVAD unloading with multiple prerequisites: ensuring an appropriate inferior vena cava diameter without hepatic congestion or RV overdistension, avoiding tricuspid insufficiency or pulmonic valve insufficiency, and maintaining a normal ratio of MAP to MPAP. Under such conditions, we were able to allow as much blood as possible to flow through the RV. Furthermore, efforts were focused on calculating the flow ratio between the RVAD's graft and the PA. The principle is that, as long as the RV function remains normal, a lower flow ratio is preferred.

Our BiVAD speed regulation involves 2 left-sided priority measures plus 6 right-sided measures, reflecting complex RVAD unloading. The challenge is to keep a balance where unloading is neither excessive—leading to iatrogenic pulmonary hypertension, severe pulmonic valve insufficiency, and right ventricular thrombus, nor insufficient—resulting in venous congestion and tricuspid insufficiency. Maintaining such a balance is far more difficult than that with a single LVAD. Moreover, we effectively controlled the refractory complication of peritoneal lymphatic leakage through a combination of measures, including consistently administering albumin, using octreotide, and applying appropriate abdominal compression.

## Prevention of RVAD Thrombosis

In our previous cases of BiVAD implantation, we observed that all venous catheters (such as those for continuous renal-replacement therapy, extracorporeal membrane oxygenation, and CVP monitoring) developed peritubular thrombi under standard anticoagulation conditions. The “kebab skewer” effect, which occurs during catheter removal, is highly likely to dislodge these peritubular thrombi, thereby increasing the risk of pump thrombosis in the RVAD.^E2^ Our experience that various efforts were ineffective in preventing right heart pump thrombosis has led us to establish a new protocol for RVAD pump thrombosis prevention: upon RVAD implantation, all deep venous catheters are no longer placed in the systemic veins. Instead, pressure measurements are obtained from a transthoracically inserted PA or even LAP lines to guide biventricular balance and to conduct fluid administration. In this case, due to severe venous hypertension, we inserted a CVP line during the initial phase of anesthesia to allow for a stable anesthetic course while we slowly drained the ascites. However, this CVP line was removed before the RVAD was initiated.

This method prevents dislodged thrombi from being aspirated into the RVAD. Postoperatively, these catheters access proved to be convenient for both monitoring and fluid delivery; it merely required a shift from traditional concepts and practices. This modification eliminated the risk of pump thrombosis. The PAP, LAP, and AP catheters were removed once the hemodynamics were stabilized. Subsequently, blood pressure was measured using a high-frequency Doppler probe, and cardiac volume and left-right heart balance were assessed via ultrasound. Fluid balance was evaluated using intake-output charts supplemented by bioelectrical impedance analysis and dual-energy X-ray absorptiometry scans, to guide the child's nutrition and rehabilitation.

The custom-designed VAD features inflow cannulas that protrude into the LA and LV by only 1 mm and 6 mm, respectively. This design prevented both peritubular thrombosis and suction, providing a valuable basis for the future development of blood pumps and surgical methods suitable for low-weight infants.

The HeartCon Ⅱ is a centrifugal blood pump designed by a unique fully magnetically sensorless levitated principle, which eliminates Hall sensors in the stator. This principle allows further miniaturization. We are currently developing a new-generation ultra-miniature RVAD that will fully meet the requirements for right ventricular support in low-weight children. The characters of low pressure, low flow, with an even smaller anatomical space will eliminate the need for flow-restricting clamp, enabling a truly “small horse pulling a small cart” scenario. Such a breakthrough over the traditional barriers of VAD therapy may enable BiVAD to replace allogeneic HTx to a considerate extent.

## Conclusions

A specially modified fully-magnetically levitated BiVAD was implanted in a 5-year, 3-month-old girl with ESHF due to RCM. Postoperatively, the patient's circulatory status was completely normalized. She underwent a successful allogeneic heart transplantation on postoperative day 40. To our knowledge, this case represents the world's lowest body-weight pediatric BiVAD implantation for end-stage RCM using the innovative ventricular assist devices.

## Conflict of Interest Statement

The authors reported no conflicts of interest.

The *Journal* policy requires editors and reviewers to disclose conflicts of interest and to decline handling or reviewing manuscripts for which they may have a conflict of interest. The editors and reviewers of this article have no conflicts of interest.

## Supplementary Data

Video 1 The key steps of the pediatric biventricular assist devices implantation surgery. Video available at: https://www.jtcvs.org/article/S2666-2507(26)00226-9/fulltext.
